# The Importance of Considering Common Variable Immunodeficiency in Patients With Chronic Diarrhea

**DOI:** 10.7759/cureus.50556

**Published:** 2023-12-15

**Authors:** Ahmad G Ansari, Husaini S Haider Mehdi, Ariba Nasar

**Affiliations:** 1 Medicine, Jawaharlal Nehru Medical College, Aligarh Muslim University, Aligarh, IND; 2 Department of Medicine, Jawaharlal Nehru Medical College, Aligarh, IND; 3 Department of Internal Medicine, Jawaharlal Nehru Medical College, Aligarh, IND

**Keywords:** approach to chronic diarrhea, chronic diarrhea, immunodeficiency, internal medicine (general medicine), common variable immunodeficiency deficiency

## Abstract

Chronic diarrhea poses a diagnostic challenge due to its diverse etiology, encompassing various gastrointestinal disorders. This case report emphasizes the clinical significance of considering common variable immunodeficiency (CVID) as a potential underlying cause in a patient presenting with chronic diarrhea. In this case study, we describe a 36-year-old female with a 9-year history of chronic diarrhea, recurrent sinopulmonary infections, and weight loss for 3 years, where previous evaluations failed to yield a diagnosis. This case underscores the diagnostic hurdles faced by healthcare professionals, often causing a delay in identifying fewer common conditions like immunodeficiency syndromes. Early recognition of CVID is crucial, enabling timely intervention with immunoglobulin replacement therapy, markedly enhancing patients' quality of life and averting complications. This report highlights the necessity for a comprehensive evaluation of non-responsive chronic diarrhea cases and raises awareness about CVID as an essential consideration, facilitating precise diagnoses and tailored treatments.

## Introduction

Common variable immunodeficiency (CVID) is one of the prevalent primary immunodeficiencies, characterized by a wide range of symptoms and recurrent bacterial infections. Patients with CVID are at increased risk of infections, including gastrointestinal infections. Chronic diarrhea is one of the presentations of CVID, and it is often the initial symptom that leads to diagnosis. Recognizing the link between CVID and chronic diarrhea is vital. Early diagnosis can guide appropriate treatments, including immunoglobulin therapy, improving patients' quality of life and preventing complications. This case report underscores the significance of considering CVID in patients with chronic diarrhea, promoting prompt recognition and intervention.

## Case presentation

A 36-year-old female presented to our emergency department with complaints of watery stools for 9 years, recurrent respiratory tract infections for 9 years, and weight loss for 3 years. Initially, the patient developed loose motions that were watery in consistency, with a stool output of about 150 mL per episode and a frequency of four to five episodes of loose motions per day, which were associated with nocturnal diarrhea. The patient also complained of coughing and nasal stuffiness for 9 years, which was associated with expectoration for the past 4 years. The patient had also complained of fever for 9 years. The frequency of the fever varied, ranging from occurring three to four times a week to sometimes once a month. The fever was mostly associated with coughing, and there was no notable association between the fever and diarrhea. The maximum temperature recorded by the patient was 100°F sublingually. The fever was associated with an evening rise in temperature, which subsided after taking over-the-counter medications. For the past 4 to 5 years, the patient has also complained of fatigue and unintentional weight loss of 8 kg. There was a history of blood transfusions in the past 10 years due to blood loss during delivery, and she was vaccinated for hepatitis B and COVID-19 in the past. There was no significant family history, or history suggestive of pulmonary tuberculosis in any family members. There was no significant obstetrical or gynecological history.

At presentation, the patient’s blood pressure was 106/60 mmHg, pulse rate was 92 per minute, respiratory rate was 14 per minute, and the temperature recorded was 98.6°F sublingually. On a general physical examination, mild pallor was observed. There were no signs of icterus, cyanosis, clubbing, pedal edema, or lymphadenopathy. The patient’s systemic examination there was nonremarkable. On anthropometric examination, her body mass index (BMI) was 18.5 kg/m^2^, and mid-upper arm circumference was 23 cm.

**Figure 1:**
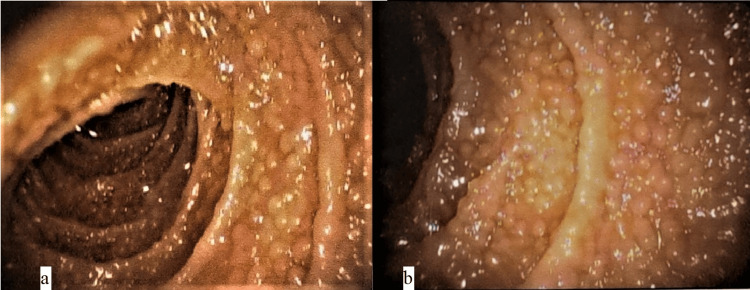
Upper GI endoscopy showing nodular lymphoid hyperplasia of the duodenum

**Figure 2:**
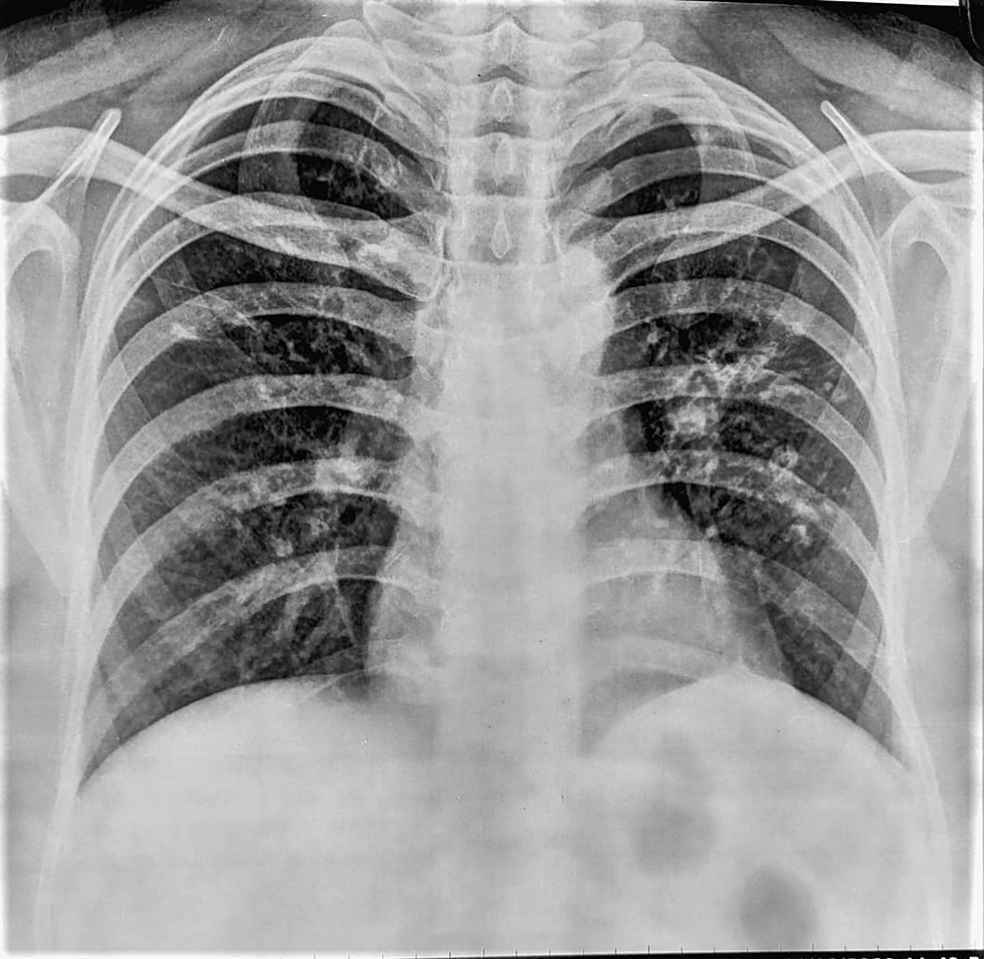
: Chest X-ray X-ray shows bilateral calcified and fibrotic lesions involving right upper, middle, and lower zones and in left upper and middle zones with evidence of hyperinflation, bronchial wall thickening in right lower zone.

Initial investigations, which were performed and are shown in Table 1, revealed mild macrocytic anemia, thrombocytopenia, hypoproteinemia with hypogammaglobinemia, vitamin B12 deficiency, folate deficiency, and vitamin D deficiency. The stool examinations were also performed with negative results for stool microscopy (for ova, cysts, and parasites), stool culture and sensitivity for pathogenic organisms, and fecal occult blood test. The patient’s calculated stool osmotic gap suggested secretory diarrhea, which corresponded with a normal 72-hour fecal fat estimation test that ruled out malabsorption syndrome and functional diarrhea (Table 2). Upper gastrointestinal endoscopy was performed, which revealed atrophic gastric mucosa and nodular lymphoid hyperplasia of the D2 part of the duodenum (Figures 1a, 1b) (Table 3). Histopathological examination of these lesions revealed chronic active duodenitis with nodular lymphoid hyperplasia with no evidence of dysplasia or malignancy. Similarly, a lower gastrointestinal endoscopy was also performed, but it was unremarkable (Table 4). Biopsies were taken from the transverse and descending colons and were also unremarkable(Table 5). An x-ray of the chest was also done and showed bilateral calcified and fibrotic lesions (Figure 2), and the sputum examination (for biochemical tests, microscopic examination for gram stain and acid-fast stain, and culture for pathogenic organisms) was nonremarkable. A Triple-Phase Contrast-Enhanced CT A whole abdomen was performed to rule out any structural disease and showed mild splenomegaly with variable-sized, homogenously enhancing mesenteric lymph nodes. A CT-guided abdominal lymph node biopsy was then performed, which showed reactive lymphocytosis with no evidence of granulomatous changes or malignancy (Table 5).

**Table 1:** Initial Investigations performed ALT: alanine aminotransferase; AST: aspartate aminotransferase

Investigation	Value	Normal Range
Hemoglobin (g/dL)	10.1	12.1-15.1
Total leucocyte count (per mL)	6200	4000-11,000
Differential leucocyte count	N65% L30% M5%	
Platelet-count(per mL)	87000	150,000-450,000
Mean corpuscular volume (femtoliters/cell)	106	80-100
Mean corpuscular hemoglobin (picogram/cell)	30	27-31
Mean corpuscular hemoglobin concentration (g/dL)	34.6	32-36
Prothrombin time (seconds)	15	11-13.5
INR	1.1	<1.1
Blood urea nitrogen (mg/dL)	12	6 to 24
Creatinine(mg/dL)	0.6	0.6-1.1
Serum sodium (mEq/L)	136	135-145
Serum potassium (mEq/L)	4.4	3.5-5.2
AST(IU/L)	20	10 to 36
ALT(IU/L)	22	19-25
Alkaline phosphatase (IU/L)	113	44-147
Total bilirubin (mg/dL)	0.63	0.1-1.2
Direct bilirubin (mg/dL)	0.23	<0.3
Total serum protein	5.2	6.7-8.6 g/dL
Serum albumin	3.2	3.5-5.5 g/dL
Serum globulin	1.2	2.0-3.5 g/dL
A/G ratio	2.6	1.5-2.5:1
Serum ferritin(ng/mL)	280	11-307
Serum iron(mcg/dL)	65	60-160
T3	152	60-215 ng/dL
T4	9.52	5.2-12.7 µg/dL
TSH	3	0.35-5.50 µIU/mL
Vitamin B12 levels (pg/mL)	170	200-900
Serum folate levels (ng/mL)	5.1	>5.38

**Table 2:** Stool examination

Stool Examination	Report	Normal Range
Microscopy for ova, cyst, and parasite	Negative	-
Stool culture and sensitivity for pathogenic organisms (aerobic and anaerobic bacterial pathogens)	Negative	-
Fecal occult blood test	Negative	-
Fecal calprotectin levels (mcg/g)	32	<50 µg/g
Fecal fat estimation (72 hours)	5.1 g/day	<7.0 g/day
Stool Giardia antigen test (Indirect Coombs test)	Negative	-
Stool Cryptosporidium antigen test (Indirect Coombs test)	Negative	-
Stool osmolality (mOsm/kg)	171	-
Stool sodium (mmol/L)	43	-
Stool potassium (mmol/L)	20	-
Stool osmotic gap (mOsm/kg)	45 mOsm/kg	Stool Osmotic Gap (Stool Osmolality - [2 * (Stool Sodium + Stool Potassium)] mOsm/kg

**Table 3:** Upper GI Endoscopy of the study patient

Upper GI Endoscopy (Figures 1a-1b)	
Esophagus	Normal
Stomach	Atrophic Gastric Mucosa
Duodenum	D1: normal; D2: multiple small, discrete, nodules scattered throughout the duodenal mucosa. The nodules are approximately 2-3 mm in diameter.

**Table 4:** Colonoscopy Report of the study patient

Procedure	Findings
Colonoscopy	The scope was passed till the transverse colon. The visualized portion did not reveal any significant abnormalities or pathological findings. The mucosa appeared healthy, and there were no visible signs of inflammation, polyps, or masses.

**Table 5:** Biopsy reports of the study patient AFB: acid-fast bacilli

Biopsy	Findings
Biopsy from duodenum	Marked villous blunting with crypt hyperplasia. Lamina propria prominent lymphoid follicles with active germinal centers and acute on chronic inflammatory infiltrate with evidence of cryptitis. Chronic active duodenitis with nodular lymphoid hyperplasia and no evidence of dysplasia/malignancy.
Biopsy from colonoscopy	Based on the histological findings, there is no evidence of pathological changes or significant abnormalities in the colonic mucosa.
Biopsy from abdominal lymph nodes including ZN (Ziehl-Neelsen) staining for AFB	The histopathological examination of the abdominal lymph node biopsy demonstrates reactive changes within the lymph nodes, characterized by enlarged germinal centers and increased lymphocyte populations with no findings suggesting any underlying granulomatous inflammation, caseation necrosis, or multinucleated giant cells and is negative on ZN staining.

A secondary clinical assessment was done to assess for chronic diarrhea associated with immunodeficiency. A serological test was conducted to detect viral markers for human immunodeficiency virus (HIV), and the result was negative. Since hypogammaglobinemia was already documented during the initial investigations, the patient was then evaluated for a poor response to immunization. The patient's anti-HBs titer was ordered, and despite receiving a booster dose of the hepatitis B vaccine in the past, low levels of anti-HBs antibodies were observed. (Table 6).

**Table 6:** Investigations performed for final diagnosis

Investigation	Value	Normal Range
HIV 1&2 antibodies(ELISA)	Negative	-
anti-HBs titers (mIU/mL)	1	>10 post-vaccination
ESR (mm/hour)	10	<20
CRP(mg/dL)	3.8	<3
Procalcitonin levels (ng/mL)	<0.1	<0.1
Immunoglobulins (mg/dL)	-	-
IgA	<33	40-350
IgG	352	650-1600
IgM	<21	50-300

Initially, the patient was given a course of injectable antibiotics for 5 days. Ceftriaxone (Gram-positive and Gram-negative coverage) and metronidazole (anaerobic coverage) were given along with supportive treatment of antisecretory agents and encouraged oral fluid intake. The patient was relieved for a few days, but her symptoms began again. After the diagnosis of CVID was considered, the patient was started on intravenous immunoglobulin (IVIG) at 500 mg/kg body weight every four weeks with premedication under supervision. The patient was discharged in satisfactory condition and was in follow-up. Serum immunoglobulin levels were done in follow-up, and they showed rising titers of serum IgA, IgG, and IgM.

During follow-up, the patient was asked about her general well-being, appetite, symptoms of cough, water stools, fever, and any complications related to IVIG administration. Two weeks after receiving the third dose of IVIG, the patient’s watery stools improved and her fever subsided. After the sixth dose, the patient's bowel movements returned to normal, her cough subsided, her appetite increased, and her BMI increased from an initial 18.5 to 21.2. The patient did not suffer from any complications from IVIG, and after the eighth dose of IVIG, the patient became asymptomatic. One year after her first dose of IVIG, the patient is still asymptomatic.

## Discussion

The patient had chronic diarrhea with recurrent sinopulmonary infections, hypogammaglobinemia, hypoproteinemia, and vitamin deficiency, along with lymphoid nodular hyperplasia of the duodenum, suggesting the diagnosis of CVID (primary immunodeficiency disorder). Other differential diagnoses that were kept were: HIV-associated enteropathy (acquired immunodeficiency disorder) as the patient had fever, recurrent respiratory tract infections, chronic diarrhea, weight loss, and a history of blood transfusion, however, HIV antibodies were negative; microscopic colitis as the patient had chronic watery diarrhea and weight loss favored the diagnosis, but the normal findings of a colon biopsy favored against it; bile salt malabsorption as the patient had chronic diarrhea, weight loss, and vitamin deficiencies favored the diagnosis; however, there was no history of abdominal pain, cramping, bloating, urgency, difficulty controlling bowel movements, or steatorrhea, which was against the diagnosis of bile salt malabsorption. Inflammatory bowel disease (IBD) was our fifth differential as the patient had chronic diarrhea, anemia, and hypoalbuminemia favored the diagnosis; however, other clinical features of IBD (blood in stools, joint pain, or other extraintestinal manifestations), histopathological evidence on biopsy, or biochemical evidence (raised ESR, CRP, and fecal calprotectin levels) were absent. Lastly, our sixth differential diagnosis was disseminated tuberculosis (pulmonary and gastrointestinal tuberculosis) as the patient had a fever with an evening rise in temperature and a chronic cough. The patient was residing in a tuberculosis-endemic region, and the patient had significant weight loss. However, there was no evidence of tuberculosis based on histopathological examination of abdominal lymph nodes and sputum, and there was no history of pain in the abdomen or alteration of bowel habits or ascites.

Chronic diarrhea is defined as the passage of abnormally liquid or unformed stools at an increased frequency with a duration of more than 4 weeks [1-2]. The approach to chronic diarrhea is different from acute diarrhea and requires complex evaluation [1]. Primary clinical assessment is done to distinguish common possibilities with further diagnostic approach requires differentiating osmotic diarrhea from secretory diarrhea by stool osmotic gap and based on this approach the study patient had secretory diarrhea [3]. Microscopic colitis presents with similar clinical features but was ruled out due to lack of histopathological evidence, similarly, bile salt diarrhea also has similar presentation but due to lack of availability of its diagnostic tests (75SeHCAT scan) in our setup and other clinical features it was not approached further. Since the patient was asymptomatic before the onset of her symptoms 9 years back, reduced immunoglobulin levels observed were likely due to acquired hypogammaglobinemia. Various causes of acquired hypogammaglobinemia are defined in the literature, namely drug-induced, infections, malignancy, and excess losses of immunoglobulins [4].

CVID is one of the primary immunodeficiency syndromes which is defined by The European Society for Immunodeficiencies (ESID) as hypogammaglobulinemia with IgG levels two standard deviations below the mean; poor vaccination responses or no isohemagglutinins; and ruling out alternative causes of hypogammaglobulinemia [5]. There is no single definitive test for CVID, and diagnosis can sometimes be challenging. The study patient was diagnosed with CVID due to her recurrent respiratory tract infection, chronic diarrhea, reduced serum IgA and IgG levels, reduced anti-HBS antibody titers, no other causes of hypogammaglobinemia were found and the disease started in the patient's second decade of life. Whole exome sequencing (WES) was performed to detect known phenotypic gene variants causing immunodeficiency and no pathognomonic or likely pathognomonic variants causative of the phenotype were detected. Prominent respiratory symptoms were also observed in study patients which could be due to recurrent respiratory tract infection or in rare cases granulomatous-lymphocytic interstitial lung disease (GLILD), which is reported in around 8-20% of cases [6]. In the large single-center prospective study done by Resnick et al. conducted in 2012, 94% of their study patients diagnosed with CVID had a history of infections; 68% of the study patients developed non-infectious complications, 29% of study patients had chronic lung disease and 15% of study patients had gastrointestinal inflammatory disease [7].

The most common infections reported in patients with CVID are bacterial infections causing sinopulmonary infections and gastrointestinal infections [8]. Apart from infections, 10-20% of patients with CVID are reported to have gastrointestinal manifestations with diarrhea being the most common symptom. These manifestations include inflammatory bowel-like disease, nodular lymphoid hyperplasia, bacterial overgrowth, nonspecific malabsorption, and gastrointestinal lymphoma [9-12]. Our patient had diffuse nodular lymphoid hyperplasia which can occur in up to 20% of CVID patients. It occurs due to chronic antigenic stimulation, these can be asymptomatic or present with pain in the abdomen, chronic diarrhea, intestinal obstruction, and, very rarely, a massive GI bleed.

Immune globulin replacement, which is the cornerstone of therapy, has significantly changed the clinical course of CVID by lowering the burden of recurrent infections and subsequent complications [13]. Early recognition and appropriate treatment not only alleviate symptoms but also enhance the overall well-being of affected individuals.

## Conclusions

Evaluation of chronic diarrhea requires thorough clinical evaluation and should be evaluated with an open mind. A review of a patient’s investigations should always be considered when clinical judgment regarding a case is not satisfied. Although common diseases should be considered first in differential diagnosis points favoring the diagnosis and points against diagnosis should be compared. CVID is a primary immunodeficiency disorder that can cause a wide range of clinical manifestations, including chronic diarrhea. Early diagnosis and treatment with immunoglobulins are essential to improve the quality of life and reduce the risk of complications in patients with CVID.
